# Metachronous multifocal early gastric cancer after partial gastrectomy for synchronous multifocal early gastric cancer: a case report and literature review

**DOI:** 10.3389/fmed.2026.1883924

**Published:** 2026-08-28

**Authors:** Qiang Geng, Qing Yang, Shubei Chen, Wei Qiu, Xiaoling Liu, Changfeng Sun, Cunliang Deng, Ou Jiang

**Affiliations:** 1 Department of Oncology, The Affiliated Hospital of Southwest Medical University, Luzhou, China; 2 The Second Department of Oncology, The Second People's Hospital of Neijiang, Neijiang, China; 3 Department of Infectious Diseases, The Affiliated Hospital of Southwest Medical University, Luzhou, China; 4 Department of Gastroenterology, The Second People's Hospital of Neijiang, Neijiang, China; 5 Department of Ultrasound, The First People's Hospital of Neijiang, Neijiang, China; 6 Department of Oncology, The First People's Hospital of Neijiang, Neijiang, China

**Keywords:** case report, endoscopic mucosal resection, endoscopic submucosal dissection, gastrectomy, metachronous gastric cancer, stomach neoplasms

## Abstract

With the rapid advancement of digestive endoscopy techniques, an increasing number of early gastric cancers (EGCs) have been diagnosed and treated, substantially improving the 5-year survival rate of patients with EGC. Multifocal gastric cancer is a relatively uncommon subtype, and repeated occurrences of multifocal gastric cancer are even less frequently observed. The pathogenesis may involve persistent field cancerization of the background gastric mucosa and, in some patients, an inherited predisposition; evaluation of mismatch repair (MMR) status is an important component of the assessment. Here, we report a young male patient who experienced two episodes of multifocal early gastric cancer over a 9-year period. Both episodes were treated with curative intent—initially by proximal one-third gastrectomy for synchronous multifocal early gastric cancer and subsequently by minimally invasive endoscopic resection for metachronous multifocal early gastric cancer in the remnant stomach. This case suggests that patients with synchronous multifocal early gastric cancer remain at risk for metachronous multifocal gastric cancer even after curative treatment. In particular, among those with high-risk profiles—including a family history of cancer, *Helicobacter pylori* infection, and gastric mucosal atrophy with intestinal metaplasia—greater emphasis should be placed on high-quality endoscopic screening, long-term surveillance, and genetic susceptibility assessment when indicated.

## Introduction

Gastric cancer is the fifth most common cancer worldwide (1). The main determinant of the 5-year survival rate is the disease stage at diagnosis. Owing to the rapid development and dissemination of endoscopic techniques, the detection of early gastric cancer has markedly increased. Endoscopic resection—including endoscopic mucosal resection (EMR) and endoscopic submucosal dissection (ESD)—has become a standard treatment for selected cases, offering advantages such as minimal invasiveness, preservation of gastric function, and improved postoperative quality of life, thereby reducing the overall medical and socioeconomic burden (2, 3). Currently, the 5-year survival rate for patients with early gastric cancer exceeds 90% (4).

Synchronous multifocal gastric cancer, most of which presents as early gastric cancer, is a relatively uncommon subtype and remains under-recognized in clinical practice. However, with the widespread adoption of magnifying endoscopy, electronic chromoendoscopy, and advanced endoscopic treatments, its detection rate has gradually increased (5). Importantly, patients with synchronous multifocal gastric cancer remain at risk of developing metachronous gastric cancer even after curative treatment. Compared with solitary gastric cancer, long-term management of patients with synchronous multifocal gastric cancer is more challenging.

In the presence of concurrent risk factors such as *H. pylori* infection, gastric mucosal atrophy with intestinal metaplasia, a family history of cancer, or a suspected genetic predisposition, meticulous endoscopic examination and long-term surveillance are essential. Herein, we report a young male patient with a family history of colorectal cancer and prior *H. pylori* infection who underwent proximal one-third gastrectomy for synchronous multifocal early gastric carcinomas. Nine years later, three metachronous early gastric cancer lesions were detected in the remnant stomach and were curatively treated by EMR and ESD. This case illustrates the occurrence of multiple primary cancers that arose at different sites within the same organ over several years and were managed with distinct treatment modalities. It also underscores the need to integrate endoscopic surveillance with timely genetic evaluation in high-risk patients.

## Case presentation

A 38-year-old male presented in 2017 with epigastric pain and underwent diagnostic gastroscopy. He had a history of *H. pylori* infection. His family history was notable for an elder brother who had died of colon cancer.

Gastroscopy revealed two ulcerative lesions located in the gastric fundus and the anterior wall of the upper gastric body, respectively. Targeted biopsies showed high-grade intraepithelial neoplasia. After multidisciplinary discussion, the patient underwent resection of the upper third of the stomach (proximal one-third gastrectomy). Postoperative pathological examination demonstrated two spatially separate lesions with intervening normal gastric mucosa: one in the gastric fundus approximately 8 mm in diameter, and the other in the anterior wall of the upper gastric body approximately 10 mm in diameter. Histopathological examination of both lesions showed high-grade intraepithelial neoplasia. No lymphovascular invasion, perineural invasion, or lymph node metastasis was identified, and there was no extension into surrounding tissues. Resection margins were negative. Both lesions were classified as high-grade intraepithelial neoplasia (HGIN), corresponding to carcinoma in situ (pTis); the final pathological stage was pTisN0M0 (6, 7). In this report, consistent with contemporary clinical practice, the term ‘early gastric cancer’ is used inclusively to encompass both HGIN (carcinoma in situ) and invasive mucosal adenocarcinoma; the 2017 lesions are therefore referred to as synchronous multifocal early gastric cancer (8).

The patient recovered uneventfully after surgery and did not receive adjuvant chemotherapy. Following discharge, he underwent *H. pylori* eradication therapy, and successful eradication was confirmed by a ^14^C-urea breath test. Thereafter, he underwent annual endoscopic surveillance.

In 2026, during routine surveillance gastroscopy at a local hospital, a 0-Is type polyp was identified in the remnant stomach. Biopsy pathology revealed high-grade intraepithelial neoplasia, and the patient was referred to our hospital for further management.

**Figure 1:**
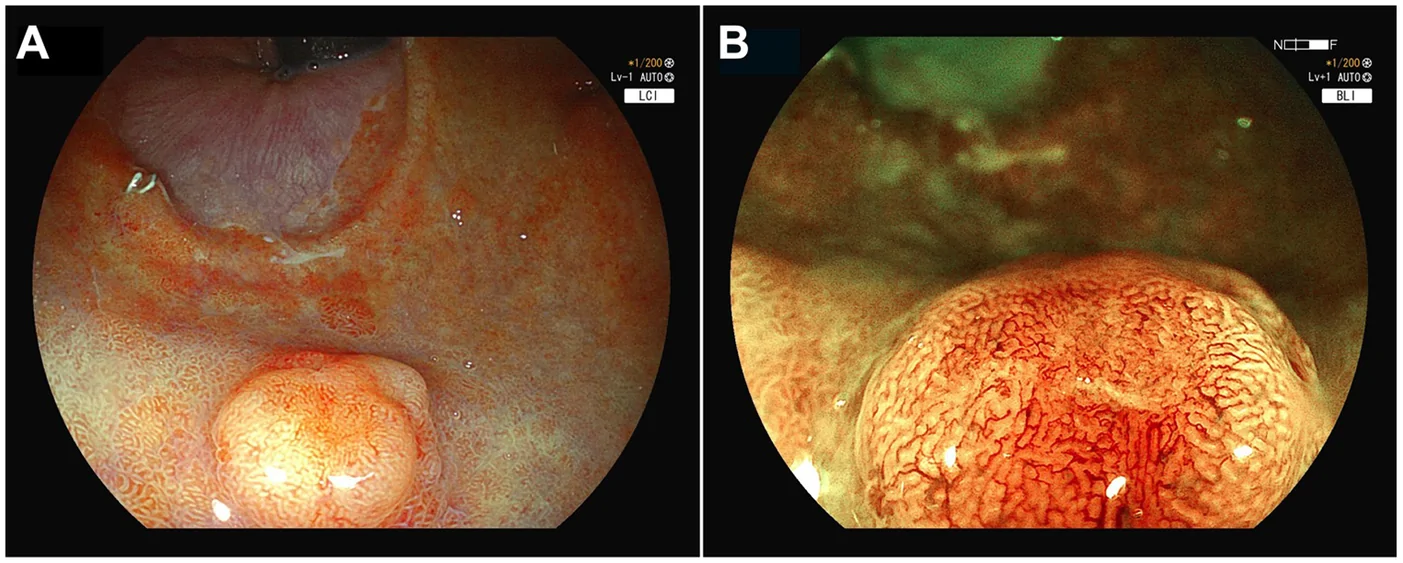
0-Is polyp on the anterior wall of the remnant stomach. **(A)** Polypoid lesion under white light imaging. **(B)** BLI+ME showing a post-biopsy scar and thickened microvessels at the tip.

**Figure 2:**
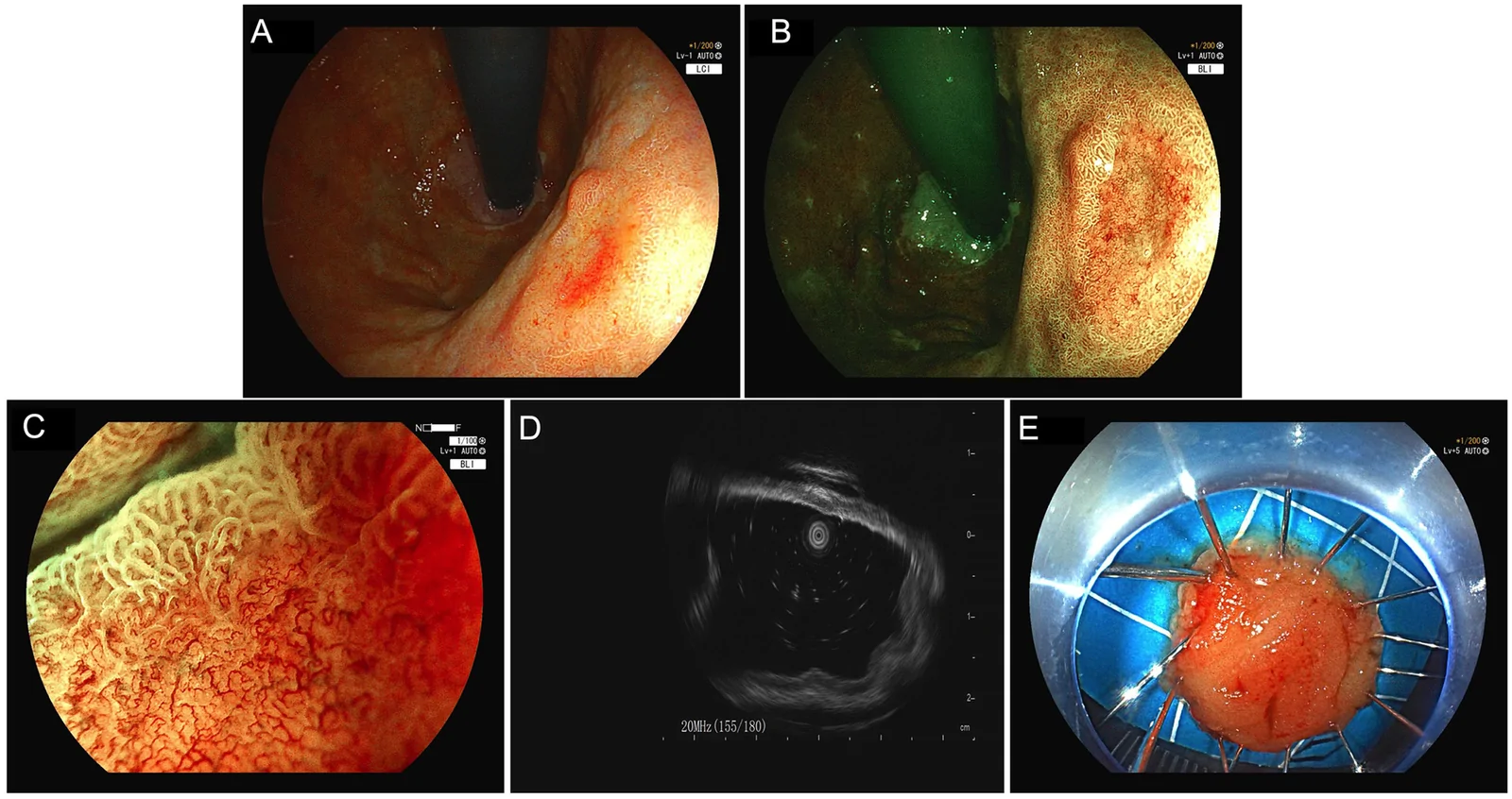
0-IIa+IIc lesion on the lesser curvature of the remnant stomach. **(A)** White light imaging shows a superficial elevation with mild central depression. **(B, C)** BLI+ME showing clear boundaries of the lesion, with irregular microvessels and surface structures visible. **(D)** EUS showing an intact muscularis mucosae. **(E)** The lesion was completely removed via ESD.

**Figure 3:**
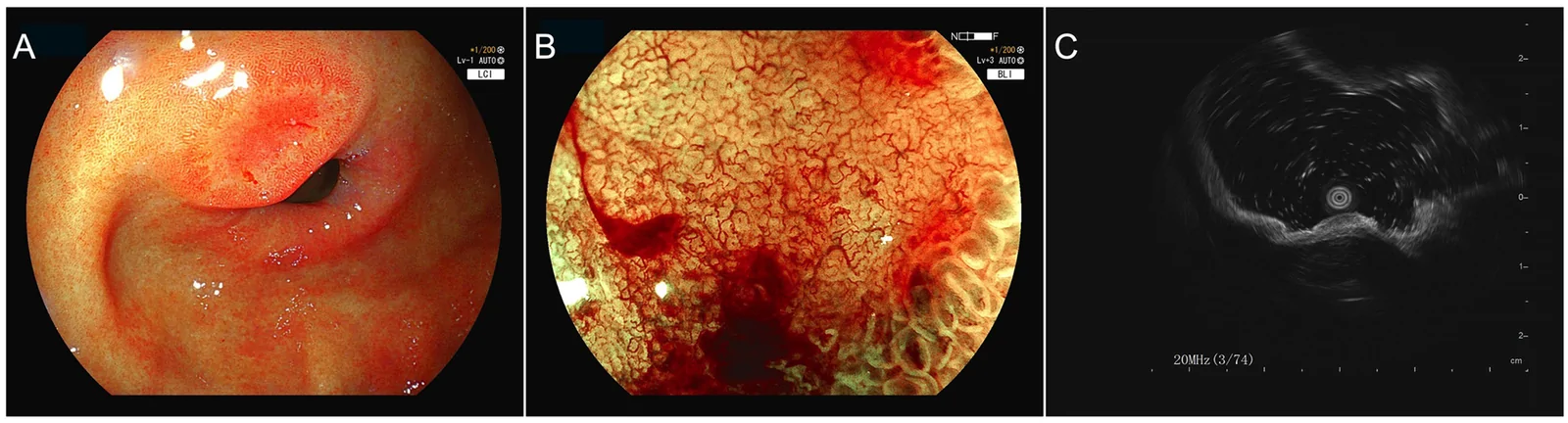
0-IIa+IIc lesion on the gastric antrum of the remnant stomach. **(A)** White light imaging shows a superficial elevation with mild central depression. **(B)** BLI+ME showing an abnormal vascular network. **(C)** EUS showing an intact muscularis mucosae.

On admission, a meticulous gastroscopic examination was performed. Three lesions were detected in the remnant stomach: (i) A 0-Is polyp measuring approximately 7 mm in diameter on the anterior wall of the remnant stomach. Under blue laser imaging (BLI) and magnifying endoscopy (ME), a post-biopsy scar and thickened microvascular structures at the tip of the polyp were observed (Figures 1A, B); (ii) A 0-IIa+IIc lesion measuring approximately 10 mm in diameter on the lesser curvature of the remnant stomach. Under BLI combined with ME, the lesion exhibited a well-demarcated border, with an irregular vascular network and abnormal glandular structures in the central area (Figures 2A–C). Endoscopic ultrasound (EUS) demonstrated an intact muscularis mucosae (Figures 2D); (iii) A 0-IIa+IIc lesion measuring approximately 6 mm in diameter on the lesser curvature of the gastric antrum. Under BLI combined with ME, an abnormal vascular network was observed (Figures 3A, B). EUS also showed an intact muscularis mucosae (Figure 3C).

Given the patient's prior history of synchronous multifocal early gastric carcinomas treated with proximal gastrectomy, the multifocal distribution of the current lesions, and the 9-year interval between the two episodes, a diagnosis of metachronous multifocal early gastric cancer was established. A ^14^C-urea breath test for *H. pylori* was negative, and contrast-enhanced abdominal computed tomography showed no evidence of lymph node or distant metastasis.

After multidisciplinary team discussion, a comprehensive review of current clinical guidelines, and consideration of the patient’s preference, we performed EMR for the lesions on the anterior wall of the remnant stomach and on the gastric antrum, and ESD for the lesion on the lesser curvature of the remnant stomach (Figure 2E).

**Figure 4:**
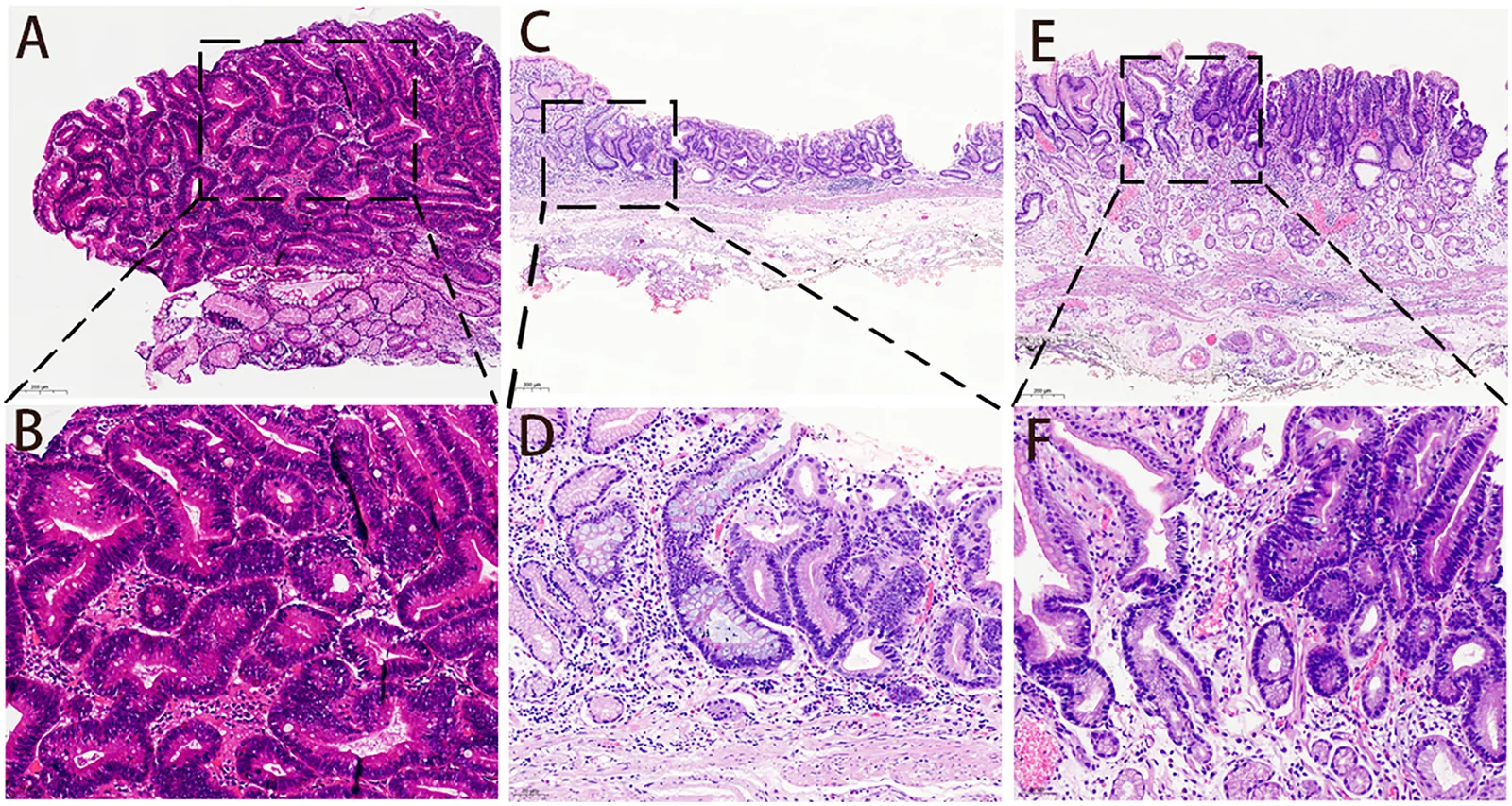
Histopathological features of three metachronous gastric lesions resected in 2026 (HE staining). All three lesions were histologically confirmed as well-differentiated tubular adenocarcinoma confined to the mucosa (pT1aN0M0). **(A, B)** Lesion on the anterior wall of the remnant stomach. **(A)** The lesion shows clear demarcation from adjacent normal mucosa, with mildly to moderately crowded neoplastic glands. Most glands retain regular contours, lined by single-layer or pseudostratified columnar cells. The nuclei are rod-shaped and hyperchromatic with evenly distributed chromatin, positioned at the base of epithelial cells with intact polarity. **(B)** High magnification of **(A)**, showing focal prominent architectural atypia characterized by irregularly branched and dilated glands. **(C, D)** Lesion on the lesser curvature of the remnant stomach. **(C)** The lesion is well demarcated from surrounding mucosa, with mildly to moderately crowded neoplastic glands. **(D)** Magnified view of **(C)** Some areas show regular glandular structures with basally located rod-shaped hyperchromatic nuclei and preserved polarity; other regions present severe architectural atypia. These atypical glands are lined by cuboidal cells with enlarged round-to-oval hyperchromatic nuclei, coarse granular chromatin and prominent nucleoli. **(E, F)** Lesion on the remnant gastric antrum. **(E)** This lesion features sharply demarcated borders and mildly crowded neoplastic glands with regular outlines, lined by single-layer or pseudostratified epithelium with basally positioned polarized rod-shaped hyperchromatic nuclei and evenly distributed chromatin. **(F)** High magnification of **(E)**, demonstrating widespread severe architectural atypia; cuboidal tumor cells contain enlarged round-to-oval hyperchromatic nuclei with coarse granular chromatin and conspicuous nucleoli. Scale bars: 200 μm **(A, C, E)**; 50 μm **(B, D, F)**.

Postoperative pathological examination revealed three lesions of well-differentiated tubular adenocarcinoma all confined to the mucosa. Horizontal and vertical margins were negative, with no lymphovascular or perineural invasion. Immunohistochemistry demonstrated proficient mismatch repair (pMMR), with intact nuclear expression of MLH1, PMS2, MSH2, and MSH6 in all three lesions. The background mucosa showed chronic atrophic gastritis with intestinal metaplasia. The final stage was pT1aN0M0 (Figures 4A–F), and all three lesions achieved curative endoscopic resection meeting eCuraA-equivalent criteria (9). The patient recovered uneventfully and was discharged in good condition. Long-term endoscopic surveillance is ongoing.

## Discussion

### Synchronous multifocal gastric cancer

Synchronous multifocal gastric cancer is defined as the presence of two or more independent tumors occurring simultaneously or within 6 months in different locations of the stomach. Each lesion must be pathologically confirmed as carcinoma, and the tumors are spatially separated by segments of histologically normal gastric wall without mutual continuity (10).

Although complete original gastroscopic images from the initial presentation are unavailable, the distribution of lesions across multiple regions of the stomach and postoperative pathology indicating multifocal, mutually independent lesions support a clinical diagnosis of synchronous multifocal gastric cancer. It should be noted that the lack of original endoscopic imaging data imposes certain retrospective limitations on this judgment.

Synchronous multifocal gastric cancers account for a relatively small proportion of all gastric cancers, but they have important clinical implications. Previous studies have reported that synchronous multifocal gastric cancers constitute approximately 6%–14% of all gastric cancers (11), although some retrospective analyses suggest that the actual proportion may be lower (12, 13). Eom et al. identified older age, male sex, family history of cancer, and tumor location in the upper third of the stomach as risk factors for synchronous multifocal gastric cancer (14). Multiple studies have demonstrated that multifocal gastric cancers predominantly present as early gastric cancer and that, compared with solitary gastric cancer, there are no significant differences in overall survival or clinicopathological parameters, including lymphovascular invasion, histologic differentiation, and lymph node metastasis (15, 16).

In the present case, multifocal lesions were identified sequentially: the 2017 lesions were HGIN (pTis), and the 2026 lesions were well-differentiated tubular adenocarcinomas confined to the mucosa; all showed favorable histology without lymphovascular, perineural, or lymph node involvement. These clinicopathological features are consistent with previous reports of multifocal gastric cancer (17, 18) and support the current consensus that minimally invasive endoscopic therapy represents an effective curative strategy for multifocal early gastric cancer with favorable histologic characteristics.

### Lessons from long-term endoscopic surveillance

One of the most critical concerns regarding synchronous multifocal gastric cancers is how to avoid missed diagnoses. Even experienced endoscopists at university-affiliated hospitals have been reported to have a diagnostic miss rate of 14.7% for synchronous multifocal gastric cancer (5). A meta-analysis showed that ESD achieves outcomes comparable to gastrectomy for early gastric cancer in terms of overall, disease-specific, and disease-free survival. However, patients treated with ESD exhibit higher incidences of recurrent, synchronous, and metachronous cancers (19). The endoscopic findings of early gastric cancer are often subtle, and the missed diagnosis rate is particularly high for small and flat lesions (20).

Therefore, once a gastric cancer lesion is detected, in addition to precise assessment of the primary lesion, the entire stomach must be systematically examined. Particular attention should be paid to perilesional regions and mucosal areas at high risk, such as those with atrophic changes or intestinal metaplasia. The use of electronic chromoendoscopy and magnifying endoscopy can improve diagnostic accuracy and help minimize missed lesions.

In our patient, multiple ulcerative lesions were identified at the initial presentation in 2017. Previous studies have reported that ulcerative gastric cancers tend to be more aggressive than non-ulcerative cancers (21), and at that time his lesions did not meet the absolute indications for endoscopic resection of early gastric cancer under the prevailing guidelines (22). Although the lesions fell within the expanded indications for endoscopic resection, endoscopic treatment was considered less favorable given the multifocal distribution and the family history of cancer; local gastric resection (proximal one-third gastrectomy) was therefore selected. Fortunately, final pathology revealed multifocal high-grade intraepithelial neoplasia, and careful long-term endoscopic follow-up subsequently detected metachronous multifocal early gastric cancers at an early stage.

### Metachronous gastric cancer and risk factors

Metachronous gastric cancer is defined as a second primary gastric cancer that occurs more than 12 months after curative treatment of the initial gastric cancer and is anatomically independent of the primary tumor. When anatomical assessment is inconclusive, molecular clonality analyses may be helpful (8, 23).

Our patient underwent regular endoscopic surveillance after the first surgery. Nine years later, three new lesions were detected in the remnant stomach. None showed direct continuity with the previous tumor sites, and imaging studies revealed no evidence of local recurrence or distant metastasis. Pathological examination supported the diagnosis of new superficial lesions. Taken together, these findings are more consistent with metachronous multifocal primary early gastric cancer than with local recurrence or residual carcinoma.

The incidence of metachronous gastric cancer typically peaks between 3 and 5 years after proximal gastrectomy, with the risk gradually decreasing beyond 10 years postoperatively (24). A nationwide Japanese survey involving 33,731 cases reported that the incidence of metachronous multiple gastric cancer after various types of gastrectomy ranges from 2.35% to 8.21%, depending on the surgical procedure (25). Another Korean study including 3,045 patients who underwent curative partial gastrectomy for gastric cancer reported a 1.1% overall incidence of metachronous gastric cancer (excluding tumors at the anastomotic site), and all cases involved solitary lesions (26). In contrast, our patient developed metachronous early gastric cancers at multiple sites in the remnant stomach, including the lesser curvature, anterior wall, and antrum. To our knowledge, this specific ‘double multifocal’ phenotype—synchronous multiple lesions at initial diagnosis followed by metachronous multiple lesions after nine years—has rarely been documented in the literature.

Established risk factors for metachronous gastric cancer include male sex, smoking, advanced age, *H. pylori* infection, gastric mucosal atrophy with intestinal metaplasia, and a history of multiple gastric cancers (27, 28). Eradication of *H. pylori* significantly reduces the risk of metachronous gastric cancer, but this benefit is attenuated in patients with severe gastric atrophy or intestinal metaplasia (29, 30). In the present case, *H. pylori* eradication was successfully achieved after the initial surgery. Therefore, the metachronous gastric cancers identified during subsequent follow-up are unlikely to be primarily attributable to persistent or recurrent *H. pylori* infection. A retrospective review of the follow-up records (Table 1) revealed that gastric mucosal atrophy was already documented one year after the initial surgery. A plausible explanation is that the patient may have already had gastric mucosal atrophy and intestinal metaplasia at the time of the initial diagnosis, although the 2017 pathology report did not specifically document the background mucosal status. These precancerous conditions, together with their associated cumulative genetic and epigenetic alterations, can persist after bacterial clearance and constitute a foundation for the long-term risk of gastric carcinogenesis.

**Table 1:** Reconstructed timeline of diagnosis, treatment, and follow-up based on available medical records.

Time	Clinical Event	Intervention	Endoscopic & Pathological Results
2017 (Initial diagnosis)	Gastroscopy performed for epigastric pain	None	Multiple ulcerative lesions in the gastric fundus and upper body. Pathology: HGIN
2017 (Treatment)	Proximal gastrectomy	Surgical resection	Postoperative diagnosis: HGIN, stage pTisN0M0
2017 (Postoperative)	*H. pylori* eradication	Quadruple therapy	Successful eradication confirmed by ^14^C-urea breath test
2018–2019	Surveillance endoscopy	Endoscopic monitoring	Atrophy of the remnant gastric antrum
2020–2023	Surveillance endoscopy	Endoscopic monitoring	Atrophy of remnant antrum and lower gastric body
2024–2025	Surveillance endoscopy	Endoscopic monitoring	Atrophy with intestinal metaplasia in remnant antrum and gastric body
2026	Surveillance endoscopy revealing a lesion	Magnifying endoscopy with treatment (EMR/ESD)	Three lesions in the remnant stomach. Pathology: well-differentiated tubular adenocarcinoma, Stage pT1aN0M0

HGIN, high-grade intraepithelial neoplasia; EMR, endoscopic mucosal resection; ESD, endoscopic submucosal dissection.

This patient had multiple high-risk factors, indicating that he should be included in a long-term high-risk management program, with intensified endoscopic surveillance and consideration of genetic evaluation.

### Treatment choice: endoscopic resection vs. completion gastrectomy

For this patient, the key therapeutic decision was between endoscopic resection and completion gastrectomy. On the one hand, the current lesions fulfilled the absolute indications for endoscopic treatment of early gastric cancer (31),and previous reports have demonstrated that ESD is feasible and effective for metachronous gastric cancer (32). On the other hand, the combination of prior synchronous multifocal gastric cancer and current metachronous multifocal lesions suggests a persistent carcinogenic susceptibility in the remnant gastric mucosa.

From a long-term risk management perspective, total remnant gastrectomy can eliminate the residual gastric mucosa and thereby reduce the future risk of further primary lesions. This approach may be particularly justifiable in young patients or when hereditary predisposition is strongly suspected. However, total gastrectomy is associated with substantial surgical morbidity, impaired nutritional status, and reduced quality of life.

After a comprehensive discussion of the potential benefits and risks of both strategies, the patient expressed a strong preference for minimally invasive therapy that preserved gastric function. Considering the small size, favorable histology, and mucosal confinement of the lesions, we regarded endoscopic treatment as an acceptable alternative, provided that intensive surveillance is maintained. Postoperative pathology confirmed curative endoscopic resection of all three lesions meeting eCuraA-equivalent criteria, and the patient remains under close endoscopic follow-up.

### Genetic evaluation in high-risk gastric cancer

Recent studies have shown that defects in MMR gene function lead to the accumulation of genomic mutations and represent a core mechanism underlying microsatellite instability-high (MSI-H) gastric cancer. MSI and MMR dysfunction are also associated with the occurrence of multiple gastric cancers (33, 34). Individuals carrying pathogenic germline variants in MMR genes (i.e., Lynch syndrome) have a markedly increased lifetime risk of developing gastric and other malignancies, including multiple primary tumors (35). MMR/MSI testing and germline genetic testing are of significant clinical importance in the precision diagnosis and treatment of gastric cancer. From an etiological perspective, MMR deficiency is closely associated with hereditary cancer syndromes, and such testing can provide valuable information regarding the tumor's molecular subtype and potential hereditary background. Furthermore, with respect to therapeutic and prognostic implications, the MSI-H/dMMR phenotype carries predictive value for responsiveness to immune checkpoint inhibitors and for long-term outcome (36). Should recurrence or metastasis develop, knowledge of the tumor's MMR/MSI status would be informative for treatment decisions.

In our patient, several clinical features raise suspicion for an underlying hereditary cancer predisposition: relatively young age at onset, a first-degree relative who died of colon cancer, and recurrent multifocal gastric cancers involving both synchronous and metachronous lesions. These features would prompt genetic counseling, tumor MMR/MSI testing, and germline multigene panel testing in contemporary clinical practice. Immunohistochemistry showed all lesions to be pMMR, reducing the likelihood of an MMR-deficient tumor pathway (including classic Lynch syndrome) as the underlying mechanism. However, pMMR does not completely exclude the possibility of germline gene mutations. Additionally, other hereditary gastric cancer syndromes cannot be excluded by MMR testing alone (37).

From a tumor biology perspective, field cancerization is the most plausible mechanism underlying multifocal gastric carcinogenesis in this case. The presence of chronic atrophic gastritis with intestinal metaplasia in the background mucosa, together with the pMMR status across all three 2026 lesions, is consistent with this interpretation—long-standing *H. pylori* infection, chronic inflammation, and environmental carcinogens may drive extensive molecular alterations that give rise to multiple independent lesions (38). Nevertheless, the relatively young age at onset and a first-degree family history of colorectal cancer remain clinical red flags for a hereditary tumor syndrome; in the absence of germline testing, a hereditary etiology cannot be excluded and warrants further molecular investigation.

In retrospect, the initial clinical management focused primarily on locoregional tumor control, while genetic evaluation was not incorporated in a timely manner. Due to the lack of MSI and germline genetic testing, accurate genetic counseling and individualized cancer risk assessment for first-degree relatives remain challenging. This case highlights that, for early-stage gastric cancer patients with high-risk features—regardless of whether the final etiology points to field cancerization or a hereditary syndrome—a comprehensive evaluation framework integrating *H. pylori* status assessment, background mucosal pathology analysis, MMR/MSI screening, and genetic counseling when indicated should be established at the time of initial diagnosis, to clarify etiology, guide long-term surveillance, and reduce risks for both the patient and at-risk family members.

#### Clinical pearls

1. Even after curative resection and successful *H. pylori* eradication, patients with synchronous multifocal early gastric cancer remain at continued risk of metachronous cancer in the remnant stomach; the surveillance window should therefore extend well beyond five years and ideally be lifelong.
2. Every surveillance endoscopy should systematically examine the entire stomach, including the remnant and anastomotic regions, with particular attention to atrophic or metaplastic mucosa; electronic chromoendoscopy and magnifying endoscopy improve detection of subtle lesions.
3. In young patients with multifocal disease and a family history of cancer, genetic counseling should be recommended regardless of tumor MMR status, because pMMR does not exclude a germline predisposition.
4. MMR immunohistochemistry can be performed on small endoscopic resection specimens and should be routinely considered when molecular characterization is clinically informative.

### Limitations

Several limitations of this case report should be acknowledged. First, the nine-year surveillance data were obtained from multiple institutions, and original endoscopic images were not available for all follow-up time points. The longitudinal clinical course was reconstructed retrospectively from available endoscopy reports. Given that standardized endoscopic examination and biopsy protocols were not uniformly applied across the participating institutions, the observed sequence of atrophy, intestinal metaplasia, and subsequent carcinogenesis should be interpreted as a suggestive observation rather than a standardized prospective natural-history documentation. Second, molecular testing was limited. The MMR status of the initial lesions could not be obtained, precluding comparison across different time points and lesions. MSI and germline genetic testing have not been completed. Germline multigene panel testing is currently unavailable at our institution, and the patient has not yet completed referral-based testing owing to financial constraints. Although the pMMR results effectively exclude somatic MMR deficiency, germline genetic testing is irreplaceable for the identification of hereditary syndromes. Third, family-history information was incomplete: the age at colorectal cancer diagnosis in the affected first-degree relative and the cancer history of other family members could not be verified from the available records, which limits the accuracy of hereditary-risk assessment.

## Conclusion

We report a case of synchronous multifocal early gastric cancer treated by proximal one-third gastrectomy, followed 9 years later by the development of metachronous multifocal early gastric cancer in the remnant stomach—a ‘double multifocal’ pattern rarely documented in the literature. The metachronous lesions were successfully treated with minimally invasive endoscopic resection, and a curative result meeting eCuraA-equivalent criteria was achieved.

This case indicates that patients with synchronous multifocal gastric cancer may remain at risk of metachronous multifocal gastric cancer during long-term follow-up, despite curative resection. For high-risk patients with a family history of cancer, a history of *H. pylori* infection, and underlying gastric mucosal atrophy with intestinal metaplasia, long-term, standardized, and meticulous endoscopic surveillance is essential. In young patients with recurrent multifocal lesions, genetic counseling and appropriate molecular testing should be strongly considered to optimize individualized management strategies for both the patient and at-risk relatives.

## Data Availability

The original contributions presented in the study are included in the article/Supplementary Material, further inquiries can be directed to the corresponding author.

## Author's note

This case report was prepared in accordance with the CARE reporting guideline for case reports; the completed CARE checklist is provided as Supplementary Material.

## References

[1] Bray F Laversanne M Sung H Ferlay J Siegel RL Soerjomataram I. Global cancer statistics 2022: GLOBOCAN estimates of incidence and mortality worldwide for 36 cancers in 185 countries. CA Cancer J Clin. (2024) 74(3):229–63. 10.3322/caac.21834 38572751

[2] Kim GH. Systematic endoscopic approach to early gastric cancer in clinical practice. Gut Liver. (2021) 15(6):811–7. 10.5009/gnl20318 33790057 PMC8593511

[3] Vasconcelos AC Dinis-Ribeiro M Libanio D. Endoscopic resection of early gastric cancer and pre-malignant gastric lesions. Cancers (Basel). (2023) 15(12):3084. 10.3390/cancers15123084 37370695 PMC10296667

[4] Conti CB Agnesi S Scaravaglio M Masseria P Dinelli ME Oldani M. Early gastric cancer: update on prevention, diagnosis and treatment. Int J Environ Res Public Health. (2023) 20(3):2149. 10.3390/ijerph20032149 36767516 PMC9916026

[5] Shi Z Zhang S Wang M Guo H Wang L Lv Y. Analysis of clinicopathologic characteristics and risk factors for missed diagnosis in synchronous multiple early gastric cancer. J Cancer Res Clin Oncol. (2025) 151(7):207. 10.1007/s00432-025-06259-x 40629181 PMC12238181

[6] Nagtegaal ID Odze RD Klimstra D Paradis V Rugge M Schirmacher P. The 2019 WHO classification of tumours of the digestive system. Histopathology. (2020) 76(2):182–8. 10.1111/his.13975 31433515 PMC7003895

[7] Amin MB, American Joint Committee on Cancer and American Cancer Society. Amin MB Edge SB Gress DM Meyer LR, editors. AJCC Cancer Staging Manual. Eight edn. Chicago IL: American Joint Committee on Cancer, Springer (2017). P. XVII, 1032.

[8] Japanese Gastric Cancer, A. Japanese Gastric cancer treatment guidelines 2025 (7th edn). Gastric Cancer. (2026) 29(2):271–99. 10.1007/s10120-025-01698-4 41569370 PMC12956939

[9] Hatta W Gotoda T Oyama T Kawata N Takahashi A Yoshifuku Y. A scoring system to stratify curability after endoscopic submucosal dissection for early gastric cancer: “eCura system”. Am J Gastroenterol. (2017) 112(6):874–81. 10.1038/ajg.2017.95 28397873

[10] Moertel CG Bargen JA Soule EH. Multiple gastric cancers; review of the literature and study of 42 cases. Gastroenterology. (1957) 32(6):1095–103. 10.1016/S0016-5085(57)80113-9 13438166

[11] Zhao B Mei D Luo R Lu H Bao S Xu H. Clinicopathological features, risk of lymph node metastasis and survival outcome of synchronous multiple early gastric cancer. Clin Res Hepatol Gastroenterol. (2020) 44(6):939–46. 10.1016/j.clinre.2020.02.004 32122791

[12] Wang X Zhou ZX Liang JW Zhang XM Meng JB. Therapeutic options and prognosis of multifocal gastric carcinomas. Zhonghua Yi Xue Za Zhi. (2009) 89(38):2705–7.PMID 20137273 20137273

[13] Jeong SH An J Kwon KA Lee WK Kim KO Chung J-W. Predictive risk factors associated with synchronous multiple early gastric cancer. Medicine (Baltimore). (2017) 96(26):e7088. 10.1097/MD.0000000000007088 28658102 PMC5500024

[14] Eom BW Lee JH Choi IJ Kook MC Nam B-H Ryu KW. Pretreatment risk factors for multiple gastric cancer and missed lesions. J Surg Oncol. (2012) 105(8):813–7. 10.1002/jso.22124 22006627

[15] Kitamura K Yamaguchi T Okamoto K Otsuji E Taniguchi H Hagiwara A. Clinicopathologic features of synchronous multifocal early gastric cancers. Anticancer Res. (1997) 17(1B):643–6. 9066594

[16] Kim HM Kim HK Lee SK Cho JH Pak KH Hyung WJ. Multifocality in early gastric cancer does not increase the risk of lymph node metastasis in a single-center study. Ann Surg Oncol. (2012) 19(4):1251–6. 10.1245/s10434-011-2083-7 22006373

[17] Zurleni T Altomare M Serio G Catalano F. Multifocal early gastric cancer in a patient with atrophic gastritis and pernicious anemia. Int J Surg Case Rep. (2020) 72:433–7. 10.1016/j.ijscr.2020.06.010 32563836 PMC7306513

[18] Zhao ZY Lai YX Xu P. Gastric ectopic pancreas combined with synchronous multiple early gastric cancer: a rare case report. World J Clin Cases. (2023) 11(7):1569–75. 10.12998/wjcc.v11.i7.1569 36926392 PMC10011978

[19] Abdelfatah MM Barakat M Ahmad D Ibrahim M Ahmed Y Kurdi Y. Long-term outcomes of endoscopic submucosal dissection versus surgery in early gastric cancer: a systematic review and meta-analysis. Eur J Gastroenterol Hepatol. (2019) 31(4):418–24. 10.1097/MEG.0000000000001352 30694909

[20] Wan J Fang Y Jiang H Wang B Xu L Hu C. Endoscopic screening for missed lesions of synchronous multiple early gastric cancer during endoscopic submucosal dissection. Gastroenterol Res Pract. (2023) 2023:2824573. 10.1155/2023/2824573 37065685 PMC10098408

[21] Lee YJ Kim J-H Park JJ Youn YH Park H Kim JW. The implications of endoscopic ulcer in early gastric cancer: can we predict clinical behaviors from endoscopy? PLoS One. (2016) 11(10):e0164339. 10.1371/journal.pone.0164339 27741275 PMC5065238

[22] Ono H Yao K Fujishiro M Oda I Nimura S Yahagi N. Guidelines for endoscopic submucosal dissection and endoscopic mucosal resection for early gastric cancer. Dig Endosc. (2016) 28(1):3–15. 10.1111/den.12518 26234303

[23] Abe S Oda I Minagawa T Sekiguchi M Nonaka S Suzuki H. Metachronous gastric cancer following curative endoscopic resection of early gastric cancer. Clin Endosc. (2018) 51(3):253–9. 10.5946/ce.2017.104 28920420 PMC5997077

[24] Kumagai K Lee S-W Ohira M Aizawa M Kamiya S Takahata T. Time interval after various types of gastrectomy until metachronous multiple gastric cancer: analysis of data from a nationwide Japanese survey. Mol Clin Oncol. (2022) 16(2):54. 10.3892/mco.2021.2487 35070303 PMC8764660

[25] Kinami S Aizawa M Yamashita H Kumagai K Kamiya S Toda M. The incidences of metachronous multiple gastric cancer after various types of gastrectomy: analysis of data from a nationwide Japanese survey. Gastric Cancer. (2021) 24(1):22–30. 10.1007/s10120-020-01104-1 32780194 PMC7790780

[26] Choi Y Kim N Yoon H Shin CM Park YS Lee DH. The incidence and risk factors for metachronous gastric cancer in the remnant stomach after gastric cancer surgery. Gut Liver. (2022) 16(3):366–74. 10.5009/gnl210202 34462394 PMC9099384

[27] Iwata Y Ito S Misawa K Ito Y Komori K Abe T. Incidence and treatment of metachronous gastric cancer after proximal gastrectomy. Surg Today. (2018) 48(5):552–7. 10.1007/s00595-018-1632-0 29460126

[28] Joo DC Kim GH. Optimal surveillance of metachronous gastric lesion after endoscopic resection of early gastric cancer. Gut Liver. (2024) 18(5):781–8. 10.5009/gnl240027 39114875 PMC11391143

[29] Fan F Wang Z Li B Zhang H. Effects of eradicating Helicobacter pylori on metachronous gastric cancer prevention: a systematic review and meta-analysis. J Eval Clin Pract. (2020) 26(1):308–15. 10.1111/jep.13179 31141285

[30] Watari J Tomita T Tozawa K Oshima T Fukui H Miwa H. Preventing metachronous gastric cancer after the endoscopic resection of gastric epithelial neoplasia: roles of *Helicobacter pylori* eradication and aspirin. Gut Liver. (2020) 14(3):281–90. 10.5009/gnl19079 31547640 PMC7234884

[31] Ono H Yao K Fujishiro M Oda I Uedo N Nimura S. Guidelines for endoscopic submucosal dissection and endoscopic mucosal resection for early gastric cancer (2nd edn). Dig Endosc. (2021) 33(1):4–20. 10.1111/den.13883 33107115

[32] Wang X Ma R Yuan L Li J Li G Ma X. Metachronous early gastric cancer successfully treated twice with endoscopic submucosal dissection: a case report. Medicine (Baltimore). (2025) 104(41):e45017. 10.1097/MD.0000000000045017 41088714 PMC12517944

[33] Shinmura K Sugimura H Naito Y Shields PG Kino I. Frequent co-occurrence of mutator phenotype in synchronous, independent multiple cancers of the stomach. Carcinogenesis. (1995) 16(12):2989–93. 10.1093/carcin/16.12.2989 8603474

[34] Ooki A Osumi H Yoshino K Yamaguchi K. Potent therapeutic strategy in gastric cancer with microsatellite instability-high and/or deficient mismatch repair. Gastric Cancer. (2024) 27(5):907–31. 10.1007/s10120-024-01523-4 38922524 PMC11335850

[35] Kanaya N Van Schaik TA Aoki H Sato Y Taniguchi F Shigeyasu K. High risk of multiple gastric cancers in Japanese individuals with lynch syndrome. Ann Gastroenterol Surg. (2024) 8(6):1008–16. 10.1002/ags3.12809 39502732 PMC11533028

[36] Le DT Uram JN Wang H Bartlett BR Kemberling H Eyring AD. PD-1 blockade in tumors with mismatch-repair deficiency. N Engl J Med. (2015) 372(26):2509–20. 10.1056/NEJMoa1500596 26028255 PMC4481136

[37] Yaghoobi M Bijarchi R Narod SA. Family history and the risk of gastric cancer. Br J Cancer. (2010) 102(2):237–42. 10.1038/sj.bjc.6605380 19888225 PMC2816643

[38] Giroux V Rustgi AK. Metaplasia: tissue injury adaptation and a precursor to the dysplasia-cancer sequence. Nat Rev Cancer. (2017) 17(10):594–604. 10.1038/nrc.2017.68 28860646 PMC5998678

