# Supplementary material for: Metachronous multifocal early gastric cancer after partial gastrectomy for synchronous multifocal early gastric cancer: a case report and literature review
Source: Front Med (Lausanne). 2026 Aug 28;13:1883924. doi: 10.3389/fmed.2026.1883924 (PMC13561814; doi:10.3389/fmed.2026.1883924)
Supplement: Supplementary file 1 [file Supplementaryfile1.pdf]

CARE
case report guidelines

CARE Checklist of information to include when writing a case report

(cc) BY-NC-ND

Topic	Item	Checklist item description	Reported on Line
Title	1	The diagnosis or intervention of primary focus followed by the words "case report"	Lines 65-73
Key Words	2	2 to 5 key words that identify diagnoses or interventions in this case report, including "case report"	Lines 108
Abstract (no references)	3a	Introduction: What is unique about this case and what does it add to the scientific literature?	Lines 85-93
	3b	Main symptoms and/or important clinical findings	Lines 93-94
	3c	The main diagnoses, therapeutic interventions, and outcomes	Lines 94-98
	3d	Conclusion—What is the main "take-away" lesson(s) from this case?	Lines 98-105
Introduction	4	One or two paragraphs summarizing why this case is unique (**may include references**)	Lines 114-136
Patient Information	5a	De-identified patient specific information.	Lines 157
	5b	Primary concerns and symptoms of the patient.	Lines 157-158
	5c	Medical, family, and psycho-social history including relevant genetic information	Lines 158-160
	5d	Relevant past interventions with outcomes	Lines 178-204
Clinical Findings	6	Describe significant physical examination (PE) and important clinical findings.	Lines 178-181
Timeline	7	Historical and current information from this episode of care organized as a timeline	Lines 205-209
Diagnostic Assessment	8a	Diagnostic testing (such as PE, laboratory testing, imaging, surveys).	Lines 210-249
	8b	Diagnostic challenges (such as access to testing, financial, or cultural)	Lines 732-758
	8c	Diagnosis (including other diagnoses considered)	Lines 304-357
	8d	Prognosis (such as staging in oncology) where applicable	Lines 357-360
Therapeutic Intervention	9a	Types of therapeutic intervention (such as pharmacologic, surgical, preventive, self-care)	Lines 298-303
	9b	Administration of therapeutic intervention (such as dosage, strength, duration)	Lines 298-303
	9c	Changes in therapeutic intervention (with rationale)	No adjustment
Follow-up and Outcomes	10a	Clinician and patient-assessed outcomes (if available)	Lines 357-358
	10b	Important follow-up diagnostic and other test results	Lines 358-360
	10c	Intervention adherence and tolerability (How was this assessed?)	Lines 360
	10d	Adverse and unanticipated events	No adverse
Discussion	11a	A scientific discussion of the strengths AND limitations associated with this case report	Lines 578-585,732-758
	11b	Discussion of the relevant medical literature **with references**.	Yes
	11c	The scientific rationale for any conclusions (including assessment of possible causes)	Lines 681-689
	11d	The primary "take-away" lessons of this case report (without references) in a one paragraph conclusion	Lines 709-729
Patient Perspective	12	The patient should share their perspective in one to two paragraphs on the treatment(s) they received	No patient perspective
Informed Consent	13	Did the patient give informed consent? Please provide if requested	Yes ☑ No ☐
