# Supplementary material for: Metachronous multifocal early gastric cancer after partial gastrectomy for synchronous multifocal early gastric cancer: a case report and literature review
Source: Front Med (Lausanne). 2026 Aug 28;13:1883924. doi: 10.3389/fmed.2026.1883924 (PMC13561814; doi:10.3389/fmed.2026.1883924)
Supplement: Supplementary file 2 [file Table1.docx]

Supplementary Fig 1.Mismatch-repair protein immunohistochemistry of the three metachronous lesions resected in 2026. All three lesions showed intact nuclear expression of MLH1, PMS2, MSH2, and MSH6 (proficient mismatch repair, pMMR). Scale bar: 50μm.
